# Lying about the Valence of Affective Pictures: An fMRI Study

**DOI:** 10.1371/journal.pone.0012291

**Published:** 2010-08-25

**Authors:** Tatia M. C. Lee, Tiffany M. Y. Lee, Adrian Raine, Chetwyn C. H. Chan

**Affiliations:** 1 Laboratory of Neuropsychology, The University of Hong Kong, Hong Kong, China; 2 Laboratory of Cognitive Affective Neuroscience, The University of Hong Kong, Hong Kong, China; 3 The State Key Laboratory of Brain and Cognitive Sciences, The University of Hong Kong, Hong Kong, China; 4 Departments of Criminology, Psychiatry, and Psychology, University of Pennsylvania, Philadelphia, Pennsylvania, United States of America; 5 Applied Cognitive Neuroscience Laboratory, Department of Rehabilitation Sciences, The Hong Kong Polytechnic University, Hong Kong, China; INSERM U901, France

## Abstract

The neural correlates of lying about affective information were studied using a functional magnetic resonance imaging (fMRI) methodology. Specifically, 13 healthy right-handed Chinese men were instructed to lie about the valence, positive or negative, of pictures selected from the International Affective Picture System (IAPS) while their brain activity was scanned by a 3T Philip Achieva scanner. The key finding is that the neural activity associated with deception is valence-related. Comparing to telling the truth, deception about the valence of the affectively positive pictures was associated with activity in the inferior frontal, cingulate, inferior parietal, precuneus, and middle temporal regions. Lying about the valence of the affectively negative pictures, on the other hand, was associated with activity in the orbital and medial frontal regions. While a clear valence-related effect on deception was observed, common neural regions were also recruited for the process of deception about the valence of the affective pictures. These regions included the lateral prefrontal and inferior parietal regions. Activity in these regions has been widely reported in fMRI studies on deception using affectively-neutral stimuli. The findings of this study reveal the effect of valence on the neural activity associated with deception. Furthermore, the data also help to illustrate the complexity of the neural mechanisms underlying deception.

## Introduction

Sometimes, we need to withhold the truth, whether for malicious or benign reasons. This act of withholding a true fact is termed “deception” [1], [2]. The involvement of brain processes in the manipulation of information suggests that brain activity during deception and truth telling should be different. It is this very assumption that founds the theoretical basis of neuroimaging studies on deception. Langleben et al. [3], Lee et al. [4], and Spence et al. [5] were among the first to apply functional magnetic resonance imaging (fMRI) methodology to deception research. Using different experimental tasks in their studies of deception, they observed that activity in the prefrontal, cingulate, and parietal regions is associated with lying (increased brain activity during lying, relative to a truth-telling control). Other studies revealed that a number of these regions are implicated in a range of cognitive processes, such as executive control, working memory, attention, and inhibition [6], [7], [8], [9], [10], [11], [12], [13]. These results, which were corroborated in later functional neuroimaging research on deception [14], [15], [16], [17], provided empirical evidence to support the insight from behavioral studies that deception is a complex and cognitively effortful task that demands a large amount of cognitive control and numerous mental management mechanisms [18].

The fMRI deception studies reviewed thus far offer insights into the brain activity associated with deception about affectively-neutral stimuli. However, little attention has been paid to the effect on brain activity when lying involves affective information. This represents a significant gap in the research, as the emotional attributes of suppressed information could have a very significant impact on deceptive responses. Recently, Abe [19] employed positron emission tomography methodology to study the neural responses associated with deception in social interactions and reported activity in the medial orbitofrontal cortex and amygdala regions. Spence et al. [20] elaborated on their previous studies [5],[17] and examined the neural activity associated with lying about past life events that the participants regarded as “embarrassing”. Lying was found to induce significant activity in the bilateral inferior frontal gyrus (Brodmann's Area [BA] 45 & 47); such activity tends to be associated with self-control and regulation [21], [22], [23]. In these studies, the deceptions were low stake. Spence et al. [24] reported the fMRI findings of a case study on deception involving a woman with possible Munchausen's syndrome by proxy who had been convicted of poisoning her child. They reported significant BOLD signals in the ventrolateral prefrontal and anterior cingulate regions. In this case study, both the content of the experimental materials used (i.e. whether she had poisoned a child) and the context of the deception (i.e. her wish to prove her innocence) could be emotion-provoking and high stake.

Indeed, on many social occasions, we may need to lie about the valence of a stimulus we encounter for reasons of social courtesy or in consideration of the feelings of others. Ganis et al. [15] found that distinct neural networks support different types of deception, depending, for instance, on whether a lie is well-rehearsed or spontaneously made up. Furthermore, there is abundant evidence that emotional materials of different valence are processed differently in the brain [25],[26],[27],[28],[29],[30]. Mak et al. [31], for example, reported that the regulation of positive and negative emotions involves common as well as distinct neural correlates. Specifically, they observed that the regulation of positive emotions was associated with changes to the BOLD signals in the left dorsal prefrontal regions and in the left insula, amygdala, right rolandic operculum, and lingual gyri regions. In contrast, the regulation of negative emotions was associated with brain activity in the left orbitofrontal gyrus, left superior frontal gyrus, anterior cingulate gyrus, left middle occipital gyrus, and right precuneus regions.

Given all of the data reviewed, the study reported here examined low-stake deception. We investigated whether lying about the valence of affective stimuli involves activity in the frontal, cingulate, and parietal regions, as has been observed in deception studies using affectively neutral stimuli. Furthermore, we examined whether lying about the valence of stimuli involves distinct neural correlates, as has been observed in the study of emotion regulation. We employed the widely used International Affective Picture System (IAPS) as the experimental stimuli [32]. The IAPS comprises a large set of standardized, emotionally evocative color photographs that are known to reliably induce emotional experiences. The stimulus set was tailored for each participant according to their rating of a set of affective pictures, which they selected from the IAPS, as either positive or negative. Orthogonal to this, the participants were cued to respond to each picture in either a truthful or a dishonest manner; for example, when cued to lie and presented with a negative picture, they had to respond that the picture was positive. Behaviorally, we would expect to perceive a typical “lie effect”, namely a significantly longer response time for the lie trials than for the truth trials. With regard to the neural correlates, we hypothesized that the regions associated with lying about positive and negative valence materials would be distinct from each other. Finally, based on the findings of the majority of imaging studies on deception, including our own work, we expected to find deception-related activations in the prefrontal cortex, anterior cingulate, and inferior parietal regions.

## Results

### Behavioral findings

#### Accuracy

The mean accuracy rate of all the participants reached over 95%. A 2-way ANOVA repeated-measures model was employed to explore the effects of the Lie versus True condition and the effect of valence perception on accuracy. Significant main effect of neither Lie/True (*F*_1,25_ = 0.59; *p* = 0.449) nor valence (*F*_1,25_ = 1.47; *p* = 0.237) was observed. The interaction effect of the two factors was also nonsignificant (*F*_1,25_ = 1.51; *p* = 0.230). The mean accuracy rate was the same for both the Lie and True conditions.

#### Reaction times

A 2-way repeated-measures ANOVA model was employed to examine the participants' reaction times. A significant main effect of the Lie and True conditions (*F*_1,25_ = 7.24; *p* = 0.013) and a robust interaction effect of Lie/True and valence perception (*F*_1,25_ = 27.49; *p*<0.001) were observed. The main effect of valence perception was nonsignificant (*F*_1,25_ = 0.71; *p*<0.406). A pair-wise comparison showed that the only significant difference between the Lie and True conditions was in relation to positive valence perception (*t* = 4.7; *p*<0.001), with a longer reaction time in the Lie (*M* = 669.09; *SD* = 157) than the True (*M* = 578.15; *SD* = 151.83) condition. The difference between the positive and negative valence was significant in both the Lie and True conditions, with a longer reaction time when participants were lying about positive (*M* = 669.09; *SD* = 157) rather than negative (*M* = 631.71; *SD* = 132.81) valence (*t* = 2.788; *p*<0.01). On the other hand, when the participants were telling the truth, they took longer to respond when they were perceiving negative (*M* = 638.78; *SD* = 133.31) rather than positive (*M* = 578.15; *SD* = 151.83) IAPS pictures, which is consistent with the findings of previous research that the processing time for stimuli charged with negative valence tends to be longer due to emotional negativity bias [33], [34], [35], [36], [37].

### Imaging results

#### Validity of the paradigm: Neural correlates of viewing the IAPS pictures

**Figure 1:**
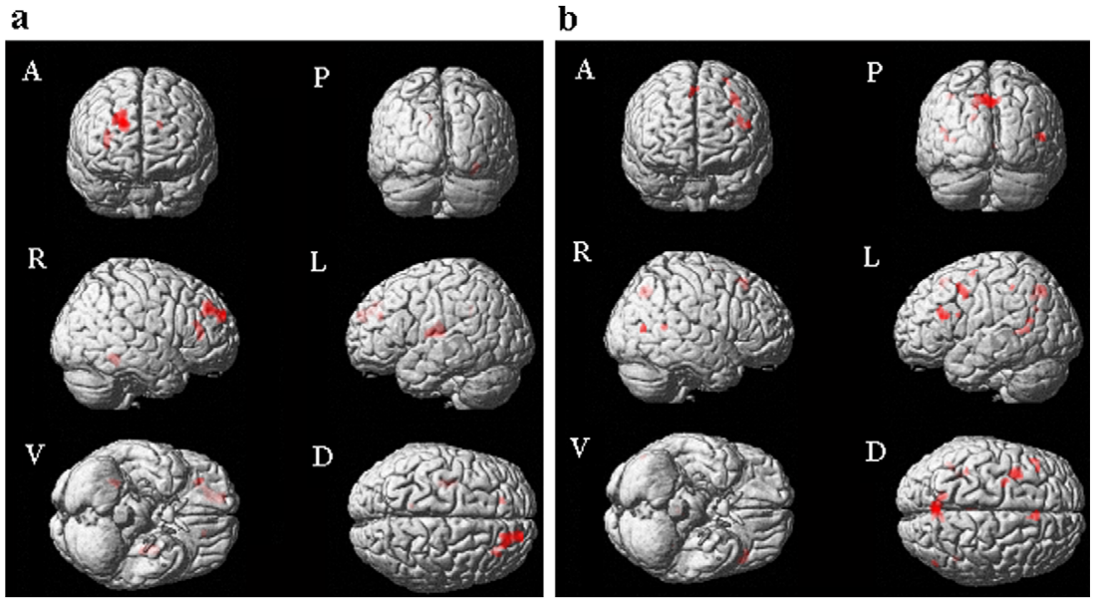
Brain activation map contrasting Positive versus Negative valence in the True condition. **1a:** True: Positive>Negative. **1b:** True: Negative>Positive. Notes: A = Anterior view; P = Posterior view; R = Right hemisphere; L = Left hemisphere; V = Ventral view; D = Dorsal view’. “True” represents the “True condition”. “Negative” represents the “Negative valence condition”; “Positive” represents the “Positive valence condition”.

By contrasting positive and negative valence in the truthful condition, we observed stronger BOLD signals in the areas of the superior frontal gyrus (BA 9), middle cingulate gyrus (BA 23), and insula (BA 45) during the perception of positive IAPS pictures; whereas the BOLD signals were stronger in the areas of the inferior frontal gyrus (BA 47 & 48), middle temporal gyrus (BA 21, 37, & 39), and anterior cingulate (BA 24) when the participants perceived negative pictures (Table 1, Figure 1). The observed activation patterns were consistent with the findings of previous studies that indicate activity in the dorsal and medial prefrontal, cingulate gyrus, and insula regions during the perception of positive emotions [38], [39], [40] and activity in the temporal gyrus, ventral prefrontal, and the anterior cingulate during the perception of affectively negative stimuli [38], [39], [41].

**Table 1:** Brain regions with significant BOLD activity in the contrasts of Positive and Negative valence in the True condition.

Regions	BA	Side	MNI Coordinates			Cluster	T
			x	y	z		
**Positive>Negative (True condition)**							
Superior Frontal	9	R	18	44	32	279	4.32
Superior Medial Frontal	32	L	−16	38	26	22	3.19
Middle Cingulum	23	L	−10	−46	36	12	2.03
Insula	45	R	34	36	10	79	2.71
Fusiform	37	R	34	−48	−12	69	2.95
Putamen	48	L	−30	−18	10	177	4.52
**Negative>Positive (True condition)**							
Superior Frontal	6	R	32	0	64	17	3.93
Superior Medial Frontal	9	R	6	48	34	27	3.4
Inferior Frontal Triangular	44	R	54	22	26	62	4.5
	45	R	54	24	0	23	3.92
Inferior Orbital Frontal	47	R	32	26	−2	45	4.88
Anterior Cingulate	−	R	8	14	24	19	3.5
Middle Cingulum	32	R	10	30	36	281	4.93
Precentral	6	L	−36	6	44	917	5.73
Supplementary Motor Area	6	L	−10	6	50	31	4.07
Inferior Parietal	40	L	−38	−40	48	159	4.39
	40	R	44	−54	48	16	4.19
Precuneus	7	L	−4	−70	44	1083	6.86
Lingual	27	R	18	−38	0	48	3.59
	19	R	24	−56	4	18	3.38
Middle Temporal	37	R	50	−70	8	79	4.7
	21	R	46	−48	12	125	4.6
Thalamus	-	R	6	−10	20	20	3.44

Notes: All of the listed brain regions were cluster corrected at 12 contiguous voxels and meet the threshold of p<0.05. The x, y, z coordinates are the Montreal Neurological Institute (MNI) coordinates. BA is the abbreviation for the approximate Brodmann's areas; L is left; R is right. Cluster Size is the number of voxels activated in the regional cluster; T is the *t*−values of the suprathreshold voxels.

#### Deception versus truth telling

The BOLD signals in the left superior medial frontal (BA 9), left inferior frontal (BA 45), left middle frontal (BA 46), left middle cingulum, bilateral insula (BA 47/48), left precentral (BA 9), bilateral inferior parietal (BA 40), left lingual (BA 37), and right thalamus were stronger for deception than truth telling. On the other hand, stronger activation was observed in the right inferior frontal (BA 6), left superior frontal (BA 6), right postcentral (BA 3 & 5), right calcarine (BA 18), and right middle occipital (BA 19) in the True condition than in the Lie condition (Table 2).

**Table 2:** Brain regions with significant BOLD activity in the contrasts of Lie and True conditions.

Regions	BA	Side	MNI Coordinates			Cluster	T
			x	y	z		
**Lie>True**							
Superior Medial Frontal	9	L	−2	56	34	1466	4.66
Inferior Frontal	45	L	−42	32	16	291	5.02
Middle Frontal	46	L	−36	26	38	31	2.7
Middle Cingulum	−	L	−16	−4	44	29	2.84
Insula	47	L	−30	30	4	211	4.64
	48	R	46	4	−4	42	2.8
Precentral	9	L	−38	10	44	47	3.11
Inferior Parietal	40	L	−40	−46	50	75	3.7
	40	R	58	−48	42	145	3.63
Lingual	37	L	−22	−42	−2	164	4.2
Thalamus	-	R	18	−26	8	38	2.69
**True>Lie**							
Superior Frontal	6	L	−16	4	52	233	3.98
Inferior Frontal	6	R	56	10	18	107	3.38
Postcentral	5	R	22	−46	66	160	2.78
	3	R	46	−18	38	28	2.54
Calcarine	18	R	18	−78	10	159	2.58
Middle Occipital	19	R	36	−70	34	174	2.51

Notes: All of the listed brain regions were cluster corrected at 12 contiguous voxels and meet the threshold of p<0.05. The x, y, z coordinates are the Montreal Neurological Institute (MNI) coordinates. BA is the abbreviation for the approximate Brodmann's areas; L is left; R is right. Cluster Size is the number of voxels activated in the regional cluster; T is the *t*-values of the suprathreshold voxels.

#### Deception of positive valence

**Figure 2:**
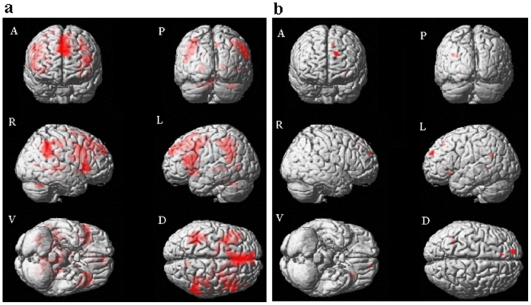
Brain activation map contrasting Lie versus True in Positive and Negative valence. **2a:** Lie>True for Positive valence. **2b:** Lie>True for Negative valence. Notes: A = Anterior view; P = Posterior view; R = Right hemisphere; L = Left hemisphere; V = Ventral view; D = Dorsal view.

Lying about the valence of the positive pictures was associated with activity in the areas of the left inferior frontal gyrus (BA 45), right middle frontal gyrus (BA 45 & 6), left middle cingulum gyrus (BA 23), right inferior parietal gyrus (BA 40), bilateral lingual (BA 19 & 30), right precuneus (BA 7), and bilateral middle temporal gyrus (BA 21 & 22). Activity in these regions has been observed in many neuroimaging studies of deception [42]. Furthermore, significant BOLD signals were observed in the visual perceptual system, the right calcarine (BA 17), and left superior occipital gyrus (BA 23) when the participants were lying about positive valence. Areas related to emotion processing, namely the left hippocampus (BA 27), left caudate, and right thalamus, were also significantly activated (Table 3, Figure 2).

**Table 3:** Brain regions with stronger BOLD activity in the Lie condition than in the True condition when perceiving Positive or Negative IAPS pictures.

Regions	BA	Side	MNI Coordinates			Cluster		T
			x	y	z			
**Lie>True (Positive valence)**								
Inferior Frontal	45	L	−42	30	14	4951		8.11
Inferior Orbital Frontal	47	R	34	24	−4	2081		4.48
Middle Frontal	45	R	50	42	18	17		3.05
	6	R	36	2	60	53		2.57
Middle Cingulum	23	L	−2	−16	28	30		2.8
Precentral	6	R	18	−16	74	12		2.19
Inferior Parietal	40	R	50	−46	40	1320		5.28
Lingual	19	L	-20	−64	2	219		3.23
	30	R	16	−42	−8	135		2.88
Precuneus	7	R	8	−62	46	74		2.66
Middle Temporal	22	L	−48	−52	24	1378		5.04
	21	R	54	−38	0	509		3.51
Superior Occipital	23	L	−20	−64	28	16		2.13
Calcarine	17	R	16	−68	8	155		2.61
Hippocampus	27	L	−22	−30	0	380		4.27
Caudate	-	L	−14	8	14	66		3.76
Thalamus	-	R	6	−6	16	56		3.63
**Lie>True (Negative valence)**								
Superior Frontal	9	L	−14	56	30		45	3.8
Superior Medial Frontal	8	L	−8	34	48		13	2.03
Inferior Orbital Frontal	47	L	−32	24	−10		15	2.44
Lingual	37	L	−22	−44	0		17	2.6

Notes: All of the listed brain regions were cluster corrected at 12 contiguous voxels and meet the threshold of p<0.05. The x, y, z coordinates are the Montreal Neurological Institute (MNI) coordinates. BA is the abbreviation for the approximate Brodmann's areas; L is left; R is right. Cluster Size is the number of voxels activated in the regional cluster; T is the *t*-values of the suprathreshold voxels.

#### Deception about negative valence

When deception about pictures of negative valence was called for, significant BOLD signals were observed in the left superior frontal gyrus (BA 9), left inferior orbital frontal gyrus (BA 47), left superior medial frontal gyrus (BA 8), and left lingual (BA 37) areas (Table 3, Figure 2).

#### Valence-related effect on deception

**Figure 3:**
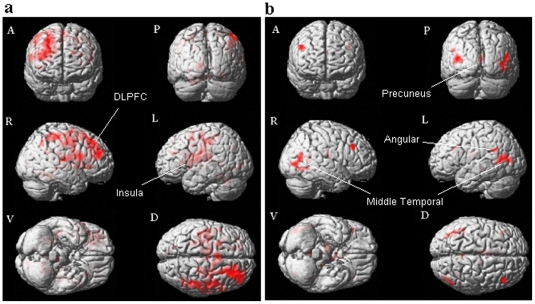
Brain activation map for the contrast of Positive versus Negative valence in the Lie condition. **3a:** Lie: Positive>Negative. **3b:** Lie: Negative>Positive. Notes: A = Anterior view; P = Posterior view; R = Right hemisphere; L = Left hemisphere; V = Ventral view; D = Dorsal view. “Negative” represents “Negative valence condition”. “Positive” represents the “Positive valence condition”. “Lie” represents the “Lie condition”. “Negative” represents the “Negative valence condition”. “Positive” represents the “Positive valence condition”.

A comparison of the BOLD signals associated with lying about positive valence, relative to that about negative valence, revealed significantly stronger signals in the bilateral middle frontal gyrus (BA 45 & 46), left insula (BA 48), left posterior cingulum (BA 29), left thalamus, left caudate, bilateral lingual gyrus (BA 18 & 19), right rolandic operculum (BA 48), right fusiform (BA 37), right superior occipital gyrus (BA 18), and left calcarine (BA 17) regions (Table 4, Figure 3). On the other hand, stronger BOLD signals were observed in the bilateral middle temporal gyrus (BA 37 & 39), bilateral middle frontal gyrus (BA 44 & 45), bilateral thalamus, left angular gyrus (BA 39), right precuneus (BA 29), left hippocampus (BA 30), and right calcarine (BA 19) when the deception about the valence was associated with negative IAPS pictures (Table 4, Figure 3). Comparing to lying about negative valence, it appears that lying about positive valence is associated with more extensive neural activity.

**Table 4:** Brain regions with significant BOLD activity in the contrasts of Positive and Negative valence conditions in the Lie condition.

Regions	BA	Side	MNI Coordinates			Cluster	T
			x	y	z		
**Positive>Negative (Lie condition)**							
Middle Frontal	45	R	46	44	20	13175	5.23
	46	L	−32	44	24	38	2.48
Insula	48	L	−32	22	8	217	4.96
Posterior Cingulum	29	L	−12	−44	10	206	3.97
Fusiform	37	R	32	−40	−20	367	3.42
Thalamus	-	L	−22	−20	0	51	3.27
Lingual	19	L	−28	−62	−2	54	3.04
	18	R	16	−76	−2	37	2.14
Calcarine	17	L	−10	−86	4	291	2.97
Rolandic Operculum	48	R	44	6	16	18	2.7
Superior Occipital	18	R	−20	−72	34	63	2.66
Caudate	-	L	−18	−26	24	15	2.28
**Negative>Positive (Lie condition)**							
Middle Temporal	37	L	−40	−68	12	332	3.89
	39	R	50	−70	14	247	3.55
Middle Frontal	45	R	44	30	32	93	3.21
Thalamus	-	L	−4	−16	20	93	3.19
	-	R	10	−16	16	80	2.77
Angular	39	L	−46	−50	28	61	3.15
Precuneus	29	R	6	−46	10	140	2.55
Hippocampus	30	L	−18	−26	−10	13	2.4
Calcarine	19	R	32	−52	10	27	2.14

Notes: All of the listed brain regions were cluster corrected at 12 contiguous voxels and meet the threshold of p<0.05. The x, y, z coordinates are the Montreal Neurological Institute (MNI) coordinates. BA is the abbreviation for the approximate Brodmann's areas; L is left; R is right. Cluster Size is the number of voxels activated in the regional cluster; T is the *t*-values of the suprathreshold voxels.

#### Common neural correlates of deception about affective stimuli

**Figure 4:**
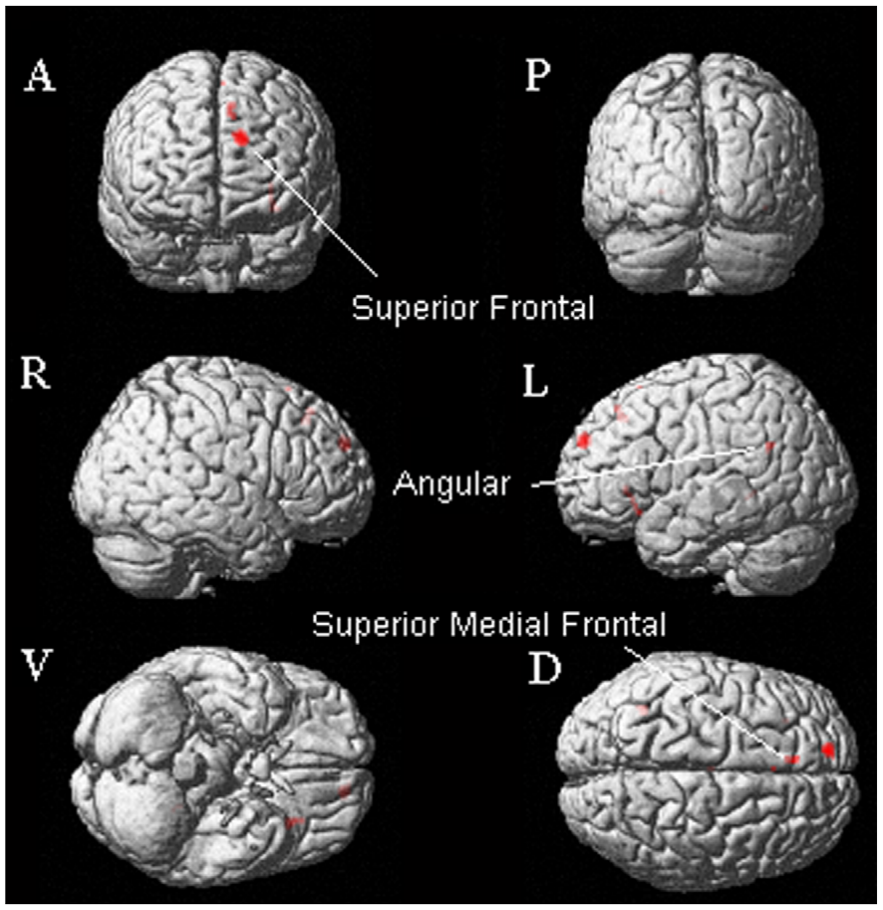
Brain activation map: conjunction analysis of contrast of Lie versus True in the Positive and the Negative valence conditions. Notes: A = Anterior view; P = Posterior view; R = Right hemisphere; L = Left hemisphere; V = Ventral view; D = Dorsal view.

We examined the neural regions in which activity was associated with deception about the valence of the affectively positive and negative pictures using the conjunction analysis procedure. We observed that the brain areas of the superior frontal gyrus (BA 9/10), superior medial frontal gyrus, inferior orbital frontal gyrus (BA 47), and angular gyrus (BA 39) were significantly activated (Table 5, Figure 4).

**Table 5:** Conjunction analysis of Lie minus True in Positive and Negative valence conditions.

Regions	BA	Side	MNI Coordinates			Cluster	T
			x	y	z		
Superior Frontal	9/10	L	−14	56	30	36	3.06
Inferior Orbital Frontal	47	L	−32	24	−10	11	2.12
Angular	39	L	−38	−56	26	12	2.07
Superior Medial Frontal	8	L	−6	34	48	15	1.99

Notes: All of the listed brain regions were cluster corrected at 10 contiguous voxels and meet the threshold of p<0.05. The x, y, z coordinates are the Montreal Neurological Institute (MNI) coordinates. BA is the abbreviation for the approximate Brodmann's areas; L is left; R is right. Cluster Size is the number of voxels activated in the regional cluster; T is the *t*-values of the suprathreshold voxels.

## Discussion

Using affectively positive and negative IAPS pictures, we studied the neural activity associated with lying about the valence of these pictures. The pattern of brain activity when the participants were viewing these IAPS pictures was consistent with that reported in previous literature, thus confirming the validity of the paradigm and hence the findings of the study.

The key finding is that the neural activity associated with deception is valence-related. In this study, compared to telling the truth, deception about the valence of the affectively positive pictures was associated with activity in the brain regions (inferior frontal region, cingulum, inferior parietal, precuneus, and middle temporal regions) commonly reported in neuroimaging studies on deception [5], [12], [16], [42], [43], [44], [45], [46], [47], [48], together with activity in the visual perceptual system (BA 17) and the limbic-related regions (the amygdala, hippocampus, thalamus, and posterior cingulate (BA 29)). Deception about the valence of negative pictures, on the other hand, was associated with activity in the orbital and medial frontal regions (BA 47 & 8), regions that are associated with emotion regulation [31], [49], [50]. A clear valence-related effect on deception was observed. Behaviorally, the lie effect, defined as the increased mean reaction time for the “lie” trials relative to the “true” trials, was significantly larger for positive pictures than for negative pictures, demonstrating a clear modulation effect of stimulus valence on deception. Together with the observed behavioral results, activity in different brain regions was involved when the participants were lying about the valence of positive (BA 7, 21, 22, 23, 40, & 45) and negative (BA 37 & 47) IAPS pictures. These results add to the existing knowledge of the role of emotion in deception [19] and, specifically, the valence-related effect on the neural activity associated with deception. The findings of this study help to illustrate the complexity of the neural mechanisms of deception.

While there is a clear valence-related effect on deception, common neural regions are also recruited (BA 8, 9, 39, & 47) during deception regardless of whether the deception involves positive or negative pictures. In other words, deception involving affective stimuli requires the collaboration of more brain regions than deception involving affectively neutral stimuli.

Deception studies that have used affectively neutral stimuli have often identified a higher level of brain activity during deception than during truth telling. This study used affective stimuli and observed that BOLD signals in the frontal-occipital regions were stronger during truth telling than during deception. This pattern of findings may relate to the participants having been engaged in a deeper level of perception during the True condition than during the Lie condition when affective stimuli were involved.

### Valence-related effect on the neural correlates of deception

#### Positive valence

Deception about positive valence was associated with activity in regions of the emotion system. For men, activity in the thalamus appears to be associated with viewing affective pictures [51], [52]. In the frontal regions, significant BOLD signals were observed in the lateral prefrontal area. Activity in these regions has been reported in previous studies on the regulation of positive emotion [31], [53]. Activity in the insula can relate to self-induced or internally generated emotions, and recalled emotions can activate the insula [54], [55]. At the same time, the insula monitors the ongoing internal emotional state of the organism [56] via extensive multi-modal sensory inputs and reciprocal connections with the cingulate gyrus, orbitofrontal cortex, and other limbic structures [57], [58].

Activity related to self-monitoring and the regulation of responses, processes which are essential to the act of deception, was also observed. Specifically, we observed significant BOLD signals in the caudate and posterior cingulum, which were also reported in our previous deception study [4]. Activity in the caudate and posterior cingulum is likely to be related to the regulation of habitual responses governed by previously learned rules and hence to monitoring the accuracy of performance [8], [59]. Semrud-Clikeman et al. [60] reported that the intact structure of the caudate correlates with the performance of measures relating to inhibition. Furthermore, Amos et al. [59] reported increased errors in performance when activation units representing striatal neurons are reduced, as observed in people with either Parkinson's or Huntington's disease. These findings suggest an important role for the caudate in the inhibition of habitual response and the monitoring of error performance associated with deceptive responses. Maguire et al. [61] proposed that the posterior cingulum links incoming information with the activated knowledge representation, hence forming an integrated representation of the discourse.

The stronger activity in the visual-perceptual system during deception about positive valence could be explained by the fact that, when engaging in deception, participants spend more time viewing positive pictures than negative pictures.

#### Negative valence

Deception about negative valence was also associated with activity in the thalamus. Again, in our previous study, activity in the thalamus was associated with emotion perception in men, regardless of the valence of the stimuli [51]. Activity in the middle frontal (BA 45) and the inferior parietal (BA 29 & 39) regions was also observed. Activity in these regions has been widely reported in imaging studies on deception by ourselves and others [4], [5], [62]. Prior research has shown that the precuneus is involved in the recollection of past events and, in particular, the retrieval of rich episodic contextual associations [63]. In this context, the higher activity we observed in the precuneus may relate to the recollection of negative affective events in the past associated with viewing the negative pictures. Activity in the limbic-related region, the hippocampus, further suggests the possibility of the recollection of an emotional memory and hence increased emotional arousal when viewing the negative pictures. The speculation regarding heightened emotional arousal is corroborated by the activity in the middle temporal and calcarine regions, which is likely to be associated with visual perception or pattern recognition associated with perceiving the IAPS pictures [64]. Although the participants were less willing to spend time viewing the negative pictures than they were viewing the positive pictures, the occipital and temporal activity was stronger when they were viewing the negative pictures. Following this line of thought, if the excessive emotional response needed to be modulated in order for the deception to be carried out, the lateral prefrontal and the inferior parietal regions may have been recruited for this purpose to provide top-down modulation [6], [22], [50], [65].

#### Valence-related effect

The patterns of the BOLD signals associated with deception about positive and negative valence appear to have been distributed in the dorsal and ventral brain regions of the emotion system, respectively. According to Phillips et al. [66], the ventral brain regions are important for the rapid appraisal of emotional material, the production of affective states, and autonomic response regulation, whereas the dorsal brain regions are important for the intentional regulation of the resulting affective states. Interpreting the imaging data in keeping with this line of thought, it appears that deception about positive valence places demands on the emotion regulation system and requires the conscious regulation of the affective state. In contrast, lying about negative valence appears to be associated with the more subconscious processes involved in the appraisal, production, and realignment of the internal affective states.

#### Common neural correlates

With regard to deceptive responses involving affective stimuli, neural activation was observed in the lateral and medial prefrontal regions and the parietal regions, which is consistent with the findings reported in prior imaging studies on deception [3], [4], [5], [12], [16]. The functional roles of these regions in relation to deception have been reported in these prior imaging studies.

### Neural correlates of deception

Taking into consideration the findings in this study using affective stimuli and in other studies using affectively neutral stimuli [67], [68], the activity in the frontal (lateral and medial) and parietal regions is robust in the act of deception, regardless of whether the stimuli used are affectively loaded or affectively neutral. On top of the neural platform of activity associated with deception, task-specific neural activity was also observed. The patterns of the BOLD signals observed during deception about the valence of the positive and negative pictures were similar to those observed in previous studies, by ourselves and others, of the emotion regulation of positive and negative emotions [31], [65], [69].

### Limitations and conclusion

The methodological limitations of this study should be acknowledged. A measure of arousal was not included in this study due to time and resource constraints. Future studies should consider investigating how arousal may modulate deception-related neural activity when the content and/or context of deception are/is affective in nature. As we used a simulated design that involved minimum stakes and induced no guilt or anxiety [3], this study could not reveal the emotional reactions involved in real-life deceptions. In addition, emotion is a multidimensional construct, and this study limited its scope to varying the valence of emotional stimuli. Other dimensions – including individual variation in the intensity of perceiving the emotional attributes of a given stimulus, the nature of the lie employed, and the emotional influence introduced by changes in the interview procedures – were not investigated. The artificial setting of the study, which involved minimum stakes when the participants lied, limits the generalizability of the findings to more applied settings. Deception in the real world is far more sophisticated than the deception in our experiments.

Notwithstanding the study's limitations, it offers some early insights into the potentially important interactions between cognition and emotion during deception. Accordingly, our findings begin to fill the theoretical gap in the current imaging research on deception, which has hitherto mainly focused on cognitive processes. Increased response time and activity in the frontal-parietal regions appear robust during deception, regardless of whether affective or affectively neutral stimuli are used. Neural activity in areas other than the frontal-parietal regions could be related to the processing of the stimuli's affective nature.

In this study, the deception-related brain activation patterns associated with materials of positive and negative valence were significantly different. This extends previous findings [15] and indicates that the neural correlates of deception depend not only on the type of lie, but also on the emotional valence of the subject content of the lie. Our findings provide an initial platform upon which future studies may build while attempting to understand the influence of emotional context on deceptive behavior. For example, it would be of theoretical and practical significance to understand the neural mechanisms of lying for rewards versus lying to avoid punishment.

## Materials and Methods

### Participants

Ethics approval was obtained from the Institutional Review Board of the University of Hong Kong/Hospital Authority Hong Kong West Cluster before the study began. All of the participants gave written informed consent for their participation. Fourteen healthy volunteers were recruited from a local community to participate in the study. All of the participants were male. Their ages ranged from 25 to 39 years (Mean age = 29.44; SD = 5.05), and, on average, they had 16.4 years of education (SD = 3.78). They were screened for any history of neurological or mental disorders and were assessed by the Edinburgh Handedness Inventory [70] as being right-handed. As one of the participants could not complete the experiment, his data were not analyzed. The findings of this study were therefore analyzed using the data sets of 13 male participants.

### Materials

Preliminary stimuli, consisting of 48 positive and 48 negative emotion-eliciting pictures, were selected from the IAPS for the participants' rating task. The pictures were chosen based on the IAPS standardized scores with matched valence and arousal levels (Positive pictures: mean valence = 7.26, SD = 0.63, mean arousal = 5.14, SD = 0.59; Negative pictures: mean valence = 3.26, SD = 0.56, mean arousal = 5.21, SD = 0.88). To minimize possible cultural differences as well as individual differences in valence perception, the final stimuli for the experimental set were selected according to each participant's ratings of the valence of the pictures. Pictures with ratings over 6 on the 9-point scale (1 = most negative; 5 = neutral; 9 = most positive) were chosen as positive valence stimuli, and pictures with ratings lower than 4 were chosen as negative valence stimuli.

### Instructions

The participants were informed that this was a study of deception. They were challenged to lie as skillfully as they could to deceive our psychologist. The participants were also informed that the experiment would involve components of both truth telling and lying and that they should pay attention to the cues of “Truth” or “Lie” which appeared before each stimulus. When the subjects were asked “How do you feel about the pictures? Positive or Negative?” (the question appeared after a stimulus), they were expected to respond as quickly as possible and in accordance with the cues. For example, if the cue was “Truth” and the picture was perceived as positive, they should respond by pressing the key designated as “Positive”. If the cue was “Lie” and the picture was perceived as positive, they should lie about the perceived valence of the picture and press the key designated as “Negative”. The response keys were counterbalanced to the participants' right index and middle fingers.

### Procedure

The participants were first given the rating task in order to select the experimental stimuli. The preliminary positive and negative IAPS pictures were presented in a randomized order for the participants to rate. Upon the presentation of each picture, the participants were prompted to rate the picture, on a 9-point scale, according to how positive/negative they felt about it (1 = most negative; 5 = neutral; 9 = most positive). To ensure the proper categorization of the positive and negative stimuli, the experimental stimuli were selected on the basis of the participants' ratings. After the rating task, the instructions for the experiment were explained to the participants, together with a brief demonstration of the experimental task. They were then given time to practice with emotionally neutral pictures in order to familiarize themselves with the speed and demands of the task.

**Figure 5:**
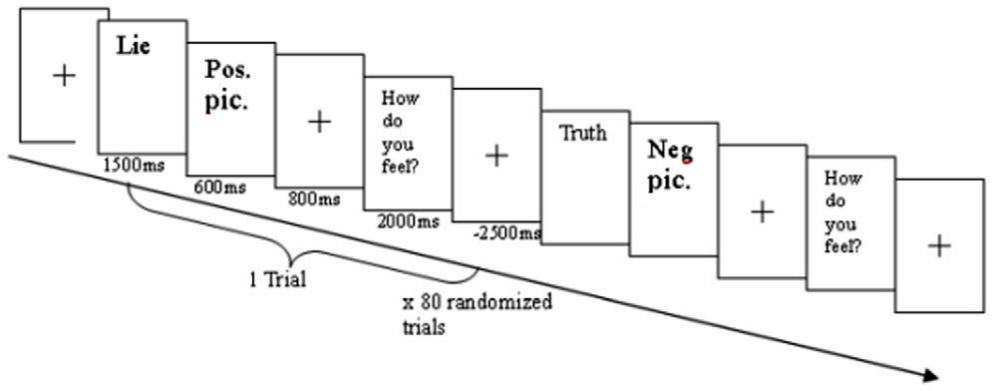
The experiment included randomized truthful and deceptive conditions. There were 20 trials in each of the four conditions: (1) True - Positive valence, (2) Lie – Positive valence, (3) True – Negative valence, and (4) Lie – Negative valence. Each trial consisted of a randomized cue, either “Truth” or “Lie”, for the True and the Lie condition, respectively. The cue appeared on the screen for 1500 ms. The participants then looked at the picture for as long as 600 ms. A fixation cross appeared for 800 ms to allow the participants to prepare to make the correct response in accordance with the cue. Then, the question “How do you feel about this picture?” was shown to prompt the participants to make either a truthful or a deceptive response according to the cue. At the end of the trial, there was a randomized jittering (average 2500 ms) to separate the trials.

An event-related design was used in the experimental paradigm. There were 80 trials involving both truthful and deceptive conditions. Half of the trials presented positive stimuli and the other half presented negative stimuli in a randomized order. As depicted in Figure 5, at the start of each trial, a visual cue (“Truth” or “Lie”) was presented for 1500 ms to indicate whether a truthful or deceptive response was required. A picture was then presented for 600 ms, and this was immediately followed by a fixation cross, which appeared for 800 ms to allow time for the participants to contemplate a response. After this, the question “How do you feel about the picture?” appeared for 2000 ms to prompt the participants to make a response in accordance with the cue. Jittering, varying from 1500 ms to 3500 ms with an average of 2500 ms, was added between each trial to allow rapid event-related randomized design. There were 40 trials for the Truth cue and 40 trials for the Lie cue. The True/Lie cues were controlled in order to combine with the Positive/Negative valence, such that a 2×2 within-subject factorial design was established, with 20 trials for each of the following conditions: (1) True Positive, (2) True Negative, (3) Lie Positive, and (4) Lie Negative.

### Image acquisition

The experiment was conducted using a 3T Philip Achieva scanner with a SENSE RF head coil with 8 channels. A T1-weighted spin-echo pulse sequence was used to acquire structural images in a sagittal orientation (TR = 7 ms, TE = 3.2 ms, Slice thickness = 1 mm), and a T2*-weighted gradient-echo echo-planar imaging pulse sequence (TR = 1800 ms, TE = 30 ms, FoV = 230 mm×230 mm, Flip angle = 90°, Slice thickness = 4 mm, Matrix dimension = 128×128) was used to acquire functional images parallel to the AC-PC plane with 32 interleaved slices.

### Data analysis

Image data were preprocessed and analyzed by the Statistical Parametric Mapping software (SPM5, Wellcome Department of Cognitive Neurology, London, UK). The functional scans of individual participants were first realigned and slice-timed to correct for temporal acquisition difference. The scans were matched onto the participants' respective structural images, which were segmented into grey/white matter. The coregistered functional scans were normalized to the SPM5 standard structural template and smoothed with an 8 mm full-width half-maximum Gaussian filter (FWHM). Hemodynamic response function (hrf) was used in modeling the signal, and a high-pass filter of 128 s was used to reduce low frequency noises. Individual contrasts among the conditions were analyzed at the first level using the fixed effect model. The results for individuals were then entered into the second level for random effect analysis. To examine differences in the neural correlates associated with the positive and negative valence conditions, we defined four regressors as follows: “Positive” refers to trials where positive valence pictures are viewed; “Negative” refers to trials in which negative valence pictures were viewed; “Lie” denotes the conditions when participants were cued to lie about the valence of the affective pictures; and “True” refers to telling the truth with regard to the perceived valence of the affective pictures. Contrasts of Positive versus Negative valence in True conditions were set up in one-sample t-tests to check the validity of the paradigm in identifying the emotional difference. Contrasts of Lie versus True in Positive and Negative valence conditions were entered into one sample t-tests separately to explore deception in relation to different emotions. A contrast on Positive versus Negative valence in Lie conditions was conducted to examine the valence-related effect on deception. Further, a conjunction analysis procedure was conducted on the contrasts of Lie versus True in both the Positive and Negative valence conditions in order to explore the neural correlates of deception common to lying about the affectively positive or negative IAPS pictures. The threshold was set at p<0.05, with a cluster correction of 12 contiguous voxels, to determine the level of statistical significance.
